# Critical protein GAPDH and its regulatory mechanisms in cancer cells

**DOI:** 10.7497/j.issn.2095-3941.2014.0019

**Published:** 2015-03

**Authors:** Jin-Ying Zhang, Fan Zhang, Chao-Qun Hong, Armando E. Giuliano, Xiao-Jiang Cui, Guang-Ji Zhou, Guo-Jun Zhang, Yu-Kun Cui

**Affiliations:** 1 Department of Physiology, Guangdong Medical College, Dongguan 523808, China; 2 Guangdong Provincial Key Laboratory for Breast Cancer Diagnosis and Treatment, Cancer Hospital of Shantou University Medical College, Shantou 515041, China; 3 Department of Surgery, Women’s Cancer Program, Samuel Oschin Comprehensive Cancer Institute, Cedars-Sinai Medical Center, Los Angeles, CA 90048, USA

**Keywords:** Glyceraldehyde-3-phosphate dehydrogenase (GAPDH), mechanism, regulation, posttranslational modification (PTM), cancer

## Abstract

Glyceraldehyde-3-phosphate dehydrogenase (GAPDH), initially identified as a glycolytic enzyme and considered as a housekeeping gene, is widely used as an internal control in experiments on proteins, mRNA, and DNA. However, emerging evidence indicates that GAPDH is implicated in diverse functions independent of its role in energy metabolism; the expression status of GAPDH is also deregulated in various cancer cells. One of the most common effects of GAPDH is its inconsistent role in the determination of cancer cell fate. Furthermore, studies have described GAPDH as a regulator of cell death; other studies have suggested that GAPDH participates in tumor progression and serves as a new therapeutic target. However, related regulatory mechanisms of its numerous cellular functions and deregulated expression levels remain unclear. GAPDH is tightly regulated at transcriptional and posttranscriptional levels, which are involved in the regulation of diverse GAPDH functions. Several cancer-related factors, such as insulin, hypoxia inducible factor-1 (HIF-1), p53, nitric oxide (NO), and acetylated histone, not only modulate *GAPDH* gene expression but also affect protein functions via common pathways. Moreover, posttranslational modifications (PTMs) occurring in GAPDH in cancer cells result in new activities unrelated to the original glycolytic function of GAPDH. In this review, recent findings related to GAPDH transcriptional regulation and PTMs are summarized. Mechanisms and pathways involved in GAPDH regulation and its different roles in cancer cells are also described.

## Introduction

Glyceraldehyde-3-phosphate dehydrogenase (GAPDH) is a glycolytic enzyme specifically catalyzing the reversible conversion of glyceraldehyde-3-phosphate (G-3-P) to 1,3-diphosphoglycerate. GAPDH participates in numerous cellular functions, in addition to glycolytic effects. For instance, GAPDH contributes to nuclear tRNA export, DNA replication and repair, endocytosis, exocytosis, cytoskeletal organization, iron metabolism, carcinogenesis, and cell death^1,2^.

Although GAPDH is widely used as an internal control, its expression status varies in different human cell lines^3^. Remarkably increased GAPDH levels are observed in many human cancer types and often correlated with reduced survival^4,5^. GAPDH is also considered as a pro-apoptotic agent^1,6^. Therefore, these deregulations of GAPDH in cancers indicate the inconsistent roles of this enzyme in cell fate determination^1,5^. However, these cancer-related mechanisms involved in GAPDH regulation remain unclear.

GAPDH is a homo tetramer containing four identical 37 kDa subunits. Localized in chromosome 12, human GAPDH gene transcribes single mRNA species, consequently producing a subunit that comprises a polypeptide chain of 335 amino acids^7,8^. GAPDH is regulated at a transcriptional level, and its functional diversity is largely affected by posttranslational modifications (PTMs) in different amino acid residues^1^. Moreover, many molecules not only regulate mRNA levels but also affect cancer-related functions (proliferation, tumor formation, chemoresistance, and so on) of GAPDH^8^. In this review, transcription-related events are described; the association of molecules with GAPDH is also discussed to determine mechanisms and pathways implicated in GAPDH regulation. Furthermore, diverse GAPDH-related PTMs, which influence the functions of this enzyme in cancer cells, are identified.

## Factors affecting *GAPDH* gene expression and protein functions

### Insulin

Early research on hepatoma cells has showed that insulin increases mRNA levels of GAPDH^9^. Further studies have revealed insulin response elements (IRE) in the upstream regulatory region of the *GAPDH* gene^10^. Among these elements, both IRE-A (−480 to −435) and IRE-B (−408 to −269) play important roles in GAPDH transcription. In a study on H35 hepatoma cells, IRE-A and IRE-B interact to enhance GAPDH transcription levels up to nearly 8-fold after insulin treatment is administered^10^. Furthermore, two insulin-sensitive DNA binding proteins (IBP) interact with these two elements^10^. These findings suggest that insulin increases GAPDH expression levels at a transcriptional level. In addition, this mechanism partially explains the overexpression of GAPDH in some cancer cells at mRNA and protein levels^11–13^.

**Figure 1:**
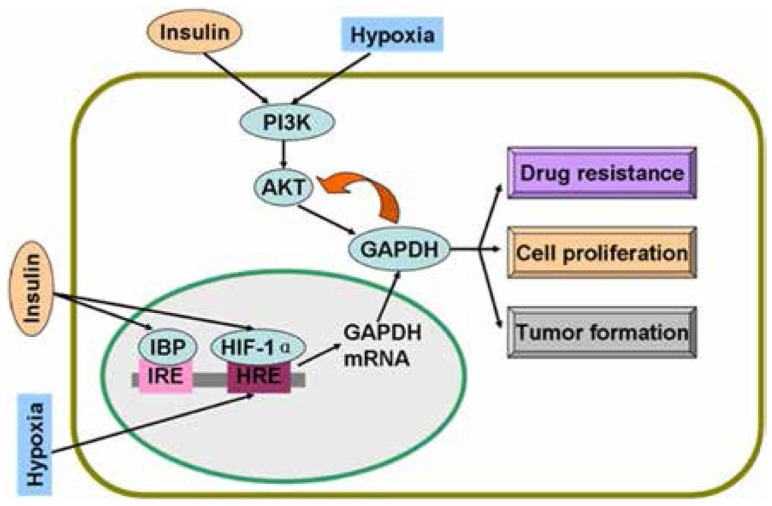
Regulatory mechanisms of GAPDH by insulin and hypoxia. Insulin and hypoxia stimulate GAPDH gene expression and activate PI3K/AKT pathway. Active AKT phosphorylates GAPDH and induces drug resistance, proliferation, and tumor formation of cancer cells. Overexpressed GAPDH interacts with active AKT and sustains enzyme activity. GAPDH, glyceraldehyde-3-phosphate dehydrogenase; IBP, insulin-binding protein; IRE, insulin response elements; HRE, hypoxia response elements; HIF-1α, hypoxia inducible factor-1α.

Studies on colon cancer cells have shown that insulin causes drug resistance and decreases chemotherapy efficacy by the activation of PI3K/AKT pathway^14,15^. Insulin is involved in the activation of AKT, a serine/threonine kinase, which phosphorylates many downstream proteins, including GAPDH^16^. AKT can phosphorylate GAPDH and enhance its glycolytic activity^16^. Interestingly, GAPDH in cancer cells interacts with active AKT and inhibits dephosphorylation; as a result, Bcl-xl is overexpressed, thereby protecting cancer cells from caspase-independent cell death (CICD)^17^. In human hepatocellular carcinoma cells, colony formation *in vitro* and tumor formation *in vivo* are decreased as GAPDH glycolytic enzyme activity is significantly decreased and phosphorylated AKT (p-AKT) is reduced when GAPDH expression is suppressed by GAPDH antagonist 3-bromopyruvate (3-BrPA) or shRNA^18^. In addition to glycolysis, GAPDH suppression decreases p-AKT and participates in tumor formation and proliferation^19^. Likewise, GAPDH inhibition caused by antisense oligonucleotides in human cervical carcinoma affects cell proliferation and induces apoptosis^20^. In addition, GAPDH is a protein target of saframycin A to decrease cancer cell proliferation^21^. Insulin-induced regulatory mechanism of GAPDH is summarized in **Figure 1**.

### Hypoxia inducible factor-1 (HIF-1)

In a study on endothelial cells, *GAPDH* gene expression is increased under hypoxia stress^22^. In another study, a hypoxia response element (HRE) is found in the *GAPDH* gene; this HRE is a 19-nucleotide sequence (−130 to −112) containing a transcription factor HIF-1 binding site^23^. The mRNA level of GAPDH is increased by approximately 75% under hypoxic condition; GAPDH overexpression is correlated with the upregulation of HIF-1α protein levels^24^. Increased HIF-1α protein levels and expression of the corresponding downstream genes encoding glycolytic enzymes are associated with mutations that activate oncogenes or inactivate tumor suppression genes^25^.

Hypoxia is a condition characterized by a decrease in oxygen level and pathophysiological condition in solid tumors^26^. Hypoxia signaling is involved in aggressive tumor behaviors^27^. In a study on lung cancer cells, hypoxia activates the PI3K/AKT pathway and induces resistance to drug-mediated apoptosis^28^. In prostate and gastric cancer cells, AKT contributes to HIF-1α expression and accumulation^29,30^. Therefore, p-AKT enhances aerobic glycolysis rate in cancer cells because p-AKT can promote the expression of glycolytic enzymes, including GAPDH, via HIF-1^31^. Furthermore, aerobic glycolysis is the main metabolic pathway of cancer cells related to cell proliferation^32^. As an important glycolytic enzyme, GAPDH participates in cancer cell proliferation^5^.

Insulin enhances HIF-1α at mRNA and protein levels in adipose tissue^33^. In a further study on pancreatic cancer, insulin stimulates HIF-1α expression under hypoxic condition; in turn, insulin requires HIF-1α to promote glycolysis and cell proliferation^34^. With HIF-1α, insulin regulates glucokinase gene expression via the PI3K/AKT pathway^35^. **Figure 1** shows the regulatory mechanisms of GAPDH by hypoxia and insulin.

### p53

mRNA and protein levels of GAPDH are upregulated by p53^36^. In a study on cytosine arabinoside-induced apoptosis, mRNA and protein levels of GAPDH are increased after p53 is overexpressed; GAPDH regulation is effectively suppressed by p53 antisense oligonucleotide. Moreover, high-efficiency transfection of *p53* gene into PC12 cells results in a remarkable overexpression of not only p53 but also GAPDH^36^. These results suggest that GAPDH overexpression is an event occurring downstream of p53; however, the specific mechanism remains unclear^36^.

As a tumor suppressor, p53 induces cell death after DNA is damaged^37^. p53 is also a transcription factor that directly binds to the promoter region of *SIAH1* gene; as a result, SIAH1 expression is increased^38^. SIAH1, seven in absentia homolog 1, is an E3 ubiquitin ligase, which moves to the nucleus and facilitates the degradation of nuclear proteins; as a consequence, apoptosis is induced^39^. Hara *et al*.^40,41^ have revealed that GAPDH binds to SIAH1; once bound, GAPDH translocates to the nucleus and stabilizes SIAH1 activity; the bound GAPDH then participates in apoptosis. Thus, p53 can upregulate the mRNA transcription of GAPDH and stimulate its participation in cell death via the SIAH1-GAPDH cascade^1^. In the absence of poly A binding protein, nuclear GAPDH enhances acetylation and serine 46 phosphorylation of p53, as well as its pro-apoptotic functions^42^. Likewise, a complex with p53 is formed, and p53 expression and phosphorylation are enhanced when GAPDH translocates to the nucleus in SIAH1-dependent manner^43^. If the p53/GAPDH complex is disrupted, cell death and GAPDH-mediated p53 upregulation and phosphorylation are possibly blocked^43^. Therefore, an auto-amplifying loop may exist in p53/GAPDH-induced apoptosis. Nevertheless, this mechanism leading to apoptosis can be prevented. In a study on ovarian cancer cells, AKT2 inhibits GAPDH nuclear translocation and suppresses GAPDH-induced apoptosis^44^. In breast cancer, GAPDH nuclear accumulation is also prevented via AKT signaling^45^.

**Figure 2:**
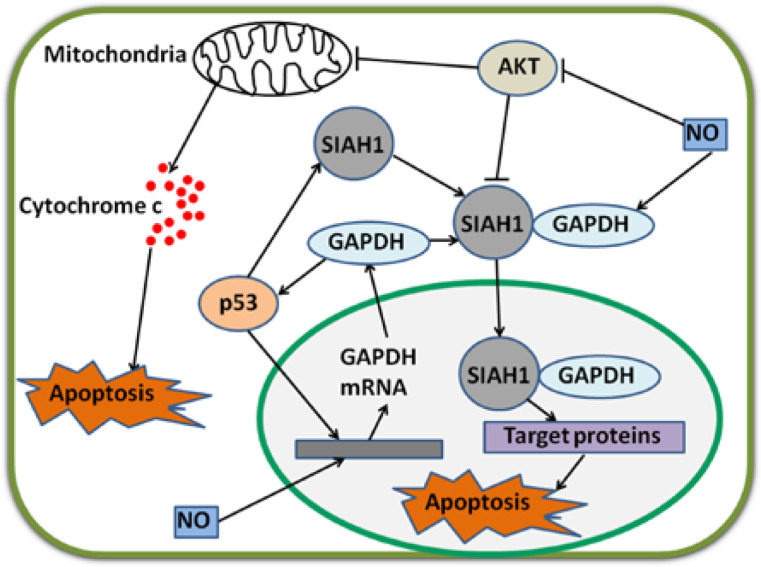
Regulatory mechanisms of GAPDH by p53 and NO. p53 and NO enhance GAPDH gene expression via unclear pathways. GAPDH enhances p53 accumulation and amplifies regulatory effect. p53 also stimulates gene expression and increases protein levels of SIAH1. Cytoplasmic GAPDH binds to SIAH1, and the bound GAPDH transports to the nucleus. SIAH1 facilitates the degradation of target proteins and consequently induces apoptosis. In this procedure, GAPDH stabilizes SIAH1 activity. NO enhances the binding ability of GAPDH to SIAH1. p53 and GAPDH stimulate mitochondrion-mediated apoptosis. Interestingly, AKT inhibits these two apoptotic pathways. NO strongly decreases phosphorylated AKT levels in cancer cells. GAPDH, glyceraldehyde-3-phosphate dehydrogenase; NO, nitric oxide.

Acetylation and phosphorylation of p53 are enhanced when GAPDH is located in the nucleus; p53 then translocates to the mitochondria to initiate apoptosis^42^. Furthermore, p53 in the mitochondria directly induces a second mitochondrion-derived caspase release, which is required for apoptosis^46^. Although AKT attenuates p53 accumulation in the mitochondria and caspase release, AKT inhibits caspase-dependent cell death^46,47^. Tarze *et al*.^48^ have reported that GAPDH also accumulates in the mitochondria and causes pro-apoptotic mitochondrial membrane permeabilization, which triggers intrinsic apoptotic pathway. In isolated mitochondria, GAPDH becomes imported, interacts with a voltage-dependent anion channel, and mediates permeability transition; as a result, the release of two pro-apoptotic proteins, namely, cytochrome C and apoptosis-inducing factor, is induced^48^. However, the regulatory mechanisms of GAPDH translocation in the mitochondria remain unclear. During apoptosis, the rapid loss of mitochondrial function is dependent on the subsequent activation of caspases promoted by the release of cytochrome C, not on mitochondrial outer membrane permeabilization (MOMP)^49^. Once caspase is inhibited, MOMP eventually leads to CICD^50^. Intriguingly, GAPDH protects cells from CICD, and this protection is dependent on an increase in glycolysis rate, nuclear translocation, and enhanced autophagy^49,51^. Furthermore, overexpressed GAPDH binds to active AKT and inhibits AKT dephosphorylation. Stabilized by GAPDH, active AKT induces phosphorylation but prevents nuclear localization of FoxO; thus, Bcl-6 levels are downregulated. Bcl-6 is a transcriptional inhibitor; a decrease in this inhibitor leads to Bcl-xL overexpression. This GAPDH-dependent increase in Bcl-xL protects the mitochondria from permeabilization; as a consequence, cells evade CICD^17^. In the nucleus, GAPDH enhances p53-mediated mitochondrion cell death; AKT inhibits GAPDH nuclear translocation and p53 mitochondrial translocation^42,46^. In the cytoplasm, overexpressed GAPDH protects tumor cells from CICD via AKT signaling pathway^17^ (**Figure 2**).

p53-induced apoptosis can be inhibited by insulin. In the kidney, high glucose induces early nephron apoptosis partially mediated by p53; conversely, insulin treatment blocks this process^52^. In human cervical carcinoma cells, insulin inhibits p53 accumulation^53^. p53 is also activated by hypoxia; moreover, p53 interacts with HIF-1α and induces ubiquitin-mediated proteosomal degradation of HIF-1α, but p53 expression is not correlated with HIF-1α expression; HIF-1α might be regulated by p53 and other factors^54,55^. In a study on colon cancer cells, HIF-1 inhibits the activation of intrinsic cell death pathway when p53 is deficient^56^. However, the combined effects of insulin, HIF-1, and p53 on GAPDH regulation remain unclear and thus warrant further studies.

### NO

In murine microvascular endothelial cells, mRNA levels of GAPDH are strongly increased after these cells are treated with TNF-α and IFN-γ; upregulation is blocked by nitric oxide (NO) synthesis inhibitors^57^. Therefore, endogenous NO can induce an increase in the mRNA expression of GAPDH^57^. Furthermore, NO modifies GAPDH (described later) and induces the translocation of GAPDH from the cytoplasm to the nucleus by enhancing the binding to SIAH1; the bound GAPDH then participates in apoptosis^58,59^. In gastric cancer cells, NO strongly decreases the levels of active AKT and suppresses cancer cell growth^60^. In ovarian cancer cells, NO donors inhibit AKT phosphorylation, prevent uncontrolled proliferation and metastasis of cancer cells, and induce cell death (**Figure 2**)^61^.

NO is produced and released from NO donors and NO synthase (NOS). NO donors increase p53 levels; NOS is transcriptionally downregulated by p53 and NOS activity is enhanced by insulin^62,63^. In solid tumors, NO inhibits hypoxia-induced chemoresistance^64^. Likewise, the combined effects of insulin, HIF-1, p53, and NO on GAPDH regulation remain obscure.

### Acetylated histone

Despite these factors, a specific molecule can downregulate gene expression and affect intracellular functions of GAPDH. In a research on glioma cells, the histone deacetylase (HDAC) inhibitor 4-phenylbutyrate (4-PB), which enhances acetylated histone, suppresses the mRNA level of GAPDH and induces apoptosis^65^. Valproic acid (VPA), another HDAC inhibitor, decreases GAPDH accumulation in the nucleus and consequently inhibits apoptosis^66^. These data confirm the inconsistent effects of HDAC inhibitors on cell fate determination; such inconsistencies are probably attributed to other cellular functions of these compounds^67^. For instance, 4-PB is described as an endoplasmic reticulum stress inhibitor, whereas VPA can induce mitochondrial damage and oxidative stress^68,69^. These processes may lead to oxidative modifications of GAPDH. Interestingly, GAPDH promotes histone acetylation. In the nucleus, S-nitrosylated GAPDH transnitrosylates HDAC. Once nitrosylated, HDAC dissociates from chromatin; as a result, histone acetylation is enhanced^70,71^. Furthermore, GAPDH can promote histone transcription. As a part of the OCA-S complex (a multicomponent Oct-1 co-activator), GAPDH binds to Oct-1 directly. In the S phase, GAPDH becomes recruited to histone-2B promoter and enhances the transcription of this promoter^72^. This result confirms the association of GAPDH with cell cycle regulation. Furthermore, overexpressed GAPDH is associated with cell cycle genes related to malignant stage and unfavorable prognosis^73^. Likewise, AKT participates in this regulatory mechanism of GAPDH via acetylated histone because AKT can stimulate histone acetylation^74^.

The regulatory mechanisms of these factors in *GAPDH* gene expression and protein functions are summarized in **Table 1**.

**Table 1:** Regulatory mechanisms of gene expression and protein function of GAPDH

Factors	mRNA level of GAPDH	GAPDH function
Insulin	Upregulating the mRNA level of GAPDH via IRE in GAPDH promoter	Enhancing glycolytic activity of GAPDH, inducing drug resistance, and protecting cells against CICD via PI3K/AKT pathway
HIF-1	Upregulating the mRNA level of GAPDH via hypoxia response element in GAPDH promoter	Enhancing glycolysis and GAPDH participation in drug resistance and cell proliferation via PI3K/AKT pathway
P53	Upregulating the mRNA level of GAPDH via an unclear mechanism	Enhancing GAPDH nuclear translocation and pro-apoptotic function, which is inhibited by AKT
NO	Upregulating the mRNA level of GAPDH via an unclear mechanism	Enhancing GAPDH nuclear translocation and pro-apoptotic function, which is inhibited by AKT
Acetylated histone	Downregulating the mRNA level of GAPDH via an unclear mechanism	Showing inconsistent effects on GAPDH cell fate that determines function via unclear mechanisms

GAPDH, glyceraldehyde-3-phosphate dehydrogenase; IRE, insulin response elements; CICD, caspase-independent cell death; NO, nitric oxide.

## PTMs of GAPDH

Possible mechanisms and pathways involved in GAPDH regulation have been discussed. GAPDH can also be regulated by other mechanisms. PTMs play important roles in regulating diverse GAPDH functions in cancer cells^1,75^.

### GAPDH phosphorylation plays inconsistent roles in cancer cells

In rat cardiac muscle, phosphorylated-GAPDH (p-GAPDH) is increased after AKT is activated^16^. In human ovarian cancer, AKT2 phosphorylates GAPDH at threonine 237 and prevents the nuclear translocation of GAPDH; as a result, the participation of GAPDH in apoptosis is impeded^44^. This result suggests that AKT-induced phosphorylation of GAPDH plays an important role in blocking cancer cell apoptosis.

In addition to AKT/PKB, protein kinase C (PKC) phosphorylates GAPDH. Tisdale *et al*. reported that PKCι/λ also phosphorylates GAPDH^76^; in this manner, GAPDH interacts with PKCι/λ and Rab2. Binding is implicated in microtubule dynamics in early secretory pathway^77,78^. Furthermore, PKCι/λ-mediated GAPDH phosphorylation is inhibited by Rab2-PKCι/λ interaction^79^. The interaction of GAPDH-PKCι/λ-Rab2 also plays an essential role in membrane recruitment and fusion^80^. GAPDH is Src-phosphorylated at tyrosine and is necessary to bind to PKCι/λ in early secretory pathway^81^. In cervical cancer, GAPDH interacts with Rab2 and participates in membrane recruitment^82^. Despite the presence of GAPDH-PKCι/λ-Rab2 in cancer cells, functions of the complex remain unclear. In addition, PKCδ can phosphorylate GAPDH in the mitochondria^83^. During ischemia and reperfusion or reoxygenation (I/R)-induced injury, GAPDH accumulates in the mitochondria, promotes the formation of lysosomal-like structures, and induces the uptake of the mitochondria into these structures^83^. However, GAPDH-driven mitophagy is possibly inhibited when PKCδ translocates to the mitochondria and phosphorylates GAPDH at threonine 246^83^. In HeLa cells, GAPDH enhances mitophagy and induces cancer cell survival^49^. PKCδ-induced phosphorylation of GAPDH may be inhibited in cancer cells to induce survival.

### S-nitrosylation of GAPDH facilitates apoptosis

GAPDH is S-nitrosylated by NO and consequently binds to SIAH1, which translocates GAPDH to the nucleus and induces cell death^41^. NO also induces S-nitrosylation of GAPDH at Cys152^84^. In HeLa cells, G-3-P partially protects GAPDH from S-nitrosylation; as a consequence, GAPDH-SIAH1 complex levels are decreased and cell survival is maintained^85^. In the cytoplasm, a novel protein interactor of GAPDH named GOSPEL (competitor of GAPDH of the SIAH1 protein enhances life) is present^86^. NO leads to S-nitrosylation of various proteins, including GAPDH and GOSPEL. Both S-nitrosylated GOSPEL and SIAH1 can bind to S-nitrosylated GAPDH; S-nitrosylated GOSPEL inhibits the binding of S-nitrosylated GAPDH to SIAH1; thus, GAPDH is retained in the cytoplasm and nuclear transport is prevented^86^. Interestingly, S-nitrosylated GAPDH is considered as a nitrosylase of nuclear proteins^87^. As such, S-nitrosylated GAPDH nitrosylates B23/nucleophosmin at cysteine 275, a binding partner of GAPDH-SIAH1 complex in the nucleus, and induces the strong binding of B23 to SIAH1; consequently, the interaction of GAPDH with SIAH1 is disrupted^88^. S-nitrosylation and nuclear translocation of GAPDH can be blocked by overexpressed glutaredoxin and CGP3466B (a drug) in non-cancer cells^89,90^. However, the effects of these inhibitors of GAPDH S-nitrosylation on human cancer cells remain obscure.

GAPDH functions as a chaperone to protect proteins from proteasomal degradation. However, GAPDH likely loses this chaperone function when GAPDH is S-nitrosylated at Cys247 by oxidatively modified low-density lipoprotein and IFNγ^91^. In cancer cells, chaperones are implicated in diverse functions, such as inducing apoptosis and enhancing proliferation^92,93^. Despite this knowledge, the roles of GAPDH as a chaperone in cancer cells remain poorly understood.

### ADP ribosylation of GAPDH promotes cell survival

GAPDH is ADP-ribosylated by transferring ADP-ribose from NAD. Arginine and cysteine are involved in modification^94–96^. ADP ribosylation of GAPDH is stimulated by S-nitrosylation of GAPDH^97,98^. In RINm5F rat tumor cells, the ADP ribosylation of GAPDH is enhanced by NO possibly because NO exhibits S-nitrosylating GAPDH function^99^. However, this ribosylation of GAPDH is stimulated by G-3-P via an unclear pathway^100^. In *Entamoeba histolytica* (*E. histolytica*), ADP-ribosylated GAPDH is found in an extracellular medium; as a result of this modification, GAPDH is incorporated in membranes, and this incorporation is the active mechanism of several bacterial toxins^101^. Thus, extracellular GAPDH from *E. histolytica* plays an essential role in interacting with host molecules and enhances the survival of this parasite in human^101^. ADP ribosylation catalyzed by poly-ADP-ribose polymerase (PARP) inactivates GAPDH enzyme activity and blocks glycolysis^102^. As glycolysis is inhibited, cells are protected from apoptosis^103^. In addition, PARP is associated with poor survival in cancer, and many PARP inhibitors as anticancer therapeutics are evaluated in several clinical trials^104,105^. Despite these findings, McDonald and Moss questioned the existence of this modification; McDonald and Moss argued that NO-stimulated, NAD-dependent modification of GAPDH is a covalent binding of the whole NAD molecule to the enzyme, not ADP ribosylation^106^.

### Carbonylation of GAPDH enhances apoptosis

In RINm5F cells, GAPDH is carbonylated by NO; as a result, GAPDH is translocated to the nucleus and apoptosis is induced, indicating that GAPDH carbonylation is implicated in cancer cell death. Furthermore, this modification is prevented by pyridoxamine and aminoguanidine^107^. These data suggest the presence of new factors that can block GAPDH-mediated apoptosis.

GAPDH can be oxidized by different sunlight components. Among these components, UV-B and γ-irradiation stimulate GAPDH carbonylation in a dose-dependent manner; UV-B and γ-irradiation also enhance the participation of GAPDH in apoptosis^108^. This mechanism may partially explain γ-irradiation therapy in cancer^109^.

### GAPDH acetylation also enhances the participation of GAPDH in apoptosis

**Figure 3:**
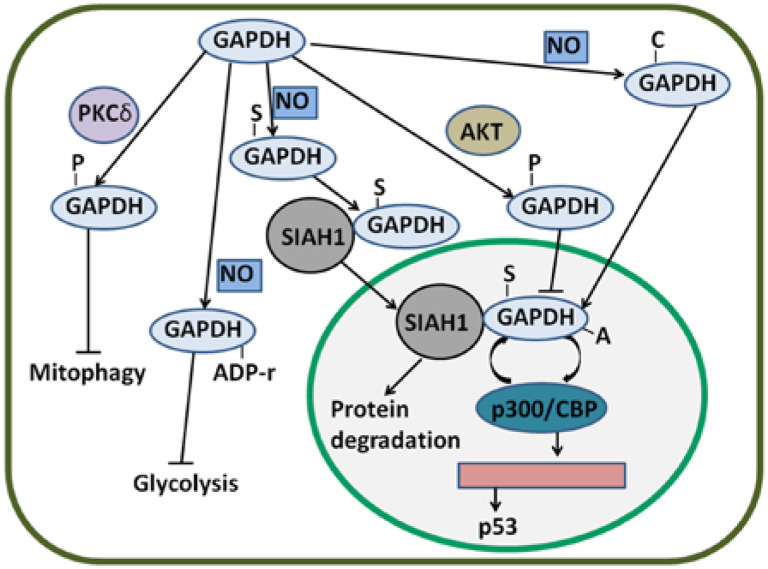
Regulatory mechanisms of GAPDH by PTMs. GAPDH can be S-nitrosylated by NO. S-nitrosylated GAPDH (S-GAPDH) then interacts with SIAH1, and the bound GAPDH translocates to the nucleus; as a result, protein degradation is induced. In the nucleus, S-GAPDH is acetylated by p300/CBP; in turn, the auto-acetylation of p300/CBP is enhanced. Activated p300/CBP stimulates p53 gene transcription. GAPDH can also be phosphorylated by AKT; thus, the nuclear translocation of GAPDH is likely inhibited. Phosphorylated GAPDH (P-GAPDH) by PKCδ in the mitochondria blocks mitophagy. NO can induce the ADP ribosylation of GAPDH and inactivate enzyme activity; as a consequence, glycolysis is inhibited. Nevertheless, NO-induced carbonylated GAPDH (C-GAPDH) can enhance nuclear translocation. GAPDH, glyceraldehyde-3-phosphate dehydrogenase; NO, nitric oxide.

Nuclear GAPDH is acetylated at Lys160 by p300 and a closely related homolog called CBP, which are among the most prominent nuclear acetyltransferases^110^. p300/CBP acetylates nuclear GAPDH, thereby enhancing the ability of GAPDH to stimulate auto-acetylation of p300/CBP. This result suggests a feed-forward activation cycle in which p300/CBP acetylates GAPDH and enhances the ability of GAPDH to stimulate the acetylation of p300/CBP; p300/CBP further acetylates GAPDH^110^. Activated p300/CBP stimulates the activation of downstream targets, including p53, and induces apoptosis^111^. p300/CBP-associated factor (PCAF) interacts with and acetylates GAPDH in three specific Lys residues (117, 227, and 251 in human cells); furthermore, PCAF mediates the transport of GAPDH from the cytoplasm to the nucleus; thus, apoptosis induced by nuclear translocation is enhanced^112^. The main PTMs of GAPDH and their effects are summarized in **Figure 3**.

Despite the PTMs of GAPDH, many other kinds of modifications are observed. For instance, Martyniuk *et al*. have showed that acrylamide forms adducts with GAPDH in Cys residues (152, 156, and 247 in human cells)^113^. These adducted formations are correlated with GAPDH enzyme inhibition. However, the roles of adducted GAPDH in cancer cells remains unclear. Protein *O*-linked *N*-acetylglucosamine acylation (O-GlcANcylation) also occurs in GAPDH. Park and his workmates have reported that O-GlcANcylation occurs at Thr227 of GAPDH in rat cells; as a result, homo tetramer formation is disrupted and the transport of GAPDH from the cytoplasm to the nucleus is induced^114^. GAPDH is also S-glutathionylated in thiol groups by S-nitrosoglutathione, a NO-releasing compound^115^. This modification affects GAPDH participation in cell death^1^. Diverse PTMs of GAPDH and their functions are summarized in **Table 2**.

**Table 2:** PTMs and their effects on GAPDH

PTMs	Effects	References
Phosphorylation	By AKT at thr237: inhibition of GAPDH nuclear translocation and pro-apoptotic functions	^44^
	By PKC ι/λ: enhancement of GAPDH participation in secretory pathway and membrane fusion	^76^
	By PKCδ: decrease in GAPDH-driven mitophagy	^83^
S-nitrosylation	By NO: stimulation of GAPDH-SIAH1 binding activity, nuclear translocation, and pro-apoptotic functions	^40^
	By NO at Cys152: stimulation of GAPDH-SIAH1 binding activity, nuclear translocation, and pro-apoptotic functions	^84^
	By IFN and oxidatively modified low-density lipoprotein at Cys247: inhibition of GAPDH chaperone functions	^91^
ADP ribosylation	Enhancement of GAPDH pro-survival functions	^101^
Carbonylation	Induced by NO: enhancement of GAPDH nuclear translocation and pro-apoptotic functions	^107^
	Induced by UV-B and γ-irradiation: enhancement of GAPDH pro-apoptotic functions	^108^
Acetylation	By p300/CBP at Lys160: enhancement of GAPDH pro-apoptotic functions	^110^
	By PCAF at Lys117, 227, and 251: enhancement of GAPDH nuclear translocation	^112^
O-GlcANcylation	Disruption of GAPDH tetramer formation and enhancement of nuclear translocation and pro-apoptotic functions	^114^
S-glutathionylation	Induced by GSNO: effect of GAPDH participation in cell death.	^115^

PTMs, posttranslational modifications; GAPDH, glyceraldehyde-3-phosphate dehydrogenase; NO, nitric oxide; PCAF, p300/CBP-associated factor.

## Conclusion

GAPDH is regulated by several cancer-related factors. Moreover, PTMs, such as phosphorylation, ADP ribosylation, and acetylation, are involved in processes by which cancer cells hijack normal pathways, as well as in cancer cell development, progression, and dissemination^105^. Indeed, glycolysis-related factors, including GAPDH, are essential for cancer cells. As a multifunctional protein, GAPDH also influences cancer cell fate. Thus, GAPDH may be a critical regulator of cancer cell functions and a marker of cancer cell progression and prognosis.
